# Prevalence of *Blastocystis* spp. and Other Gastrointestinal Pathogens Among Patients Admitted to Research Hospitals in Campania Region, Italy

**DOI:** 10.3390/pathogens14050425

**Published:** 2025-04-27

**Authors:** Marianna Ascierto, Annalisa Chianese, Francesco Foglia, Emiliana Finamore, Luciana Petrullo, Carla Zannella, Anna De Filippis, Maria Grazia Coppola, Massimiliano Galdiero

**Affiliations:** 1 Department of Experimental Medicine, University of Campania “L. Vanvitelli”, 80138 Naples, Italy; marianna.ascierto@studenti.unicampania.it (M.A.); annalisa.chianese@unicampania.it (A.C.); francesco.foglia@unicampania.it (F.F.); emiliana.finamore@unicampania.it (E.F.); carla.zannella@unicampania.it (C.Z.); anna.defilippis@unicampania.it (A.D.F.); 2 Complex Operative Unit of Virology and Microbiology, University Hospital of Campania “L. Vanvitelli”, 80138 Naples, Italy; 3 Unit of Microbiology, Cotugno Hospital, AORN “Ospedali dei Colli”, 80131 Naples, Italy; luciana.petrullo@ospedalideicolli.it (L.P.); mariagrazia.coppola@ospedalideicolli.it (M.G.C.)

**Keywords:** blastocystosis, parasites, gastrointestinal parasitic infections, enteric pathogens

## Abstract

Background. *Blastocystis* spp. is a common protozoan found in the gastrointestinal tract, typically existing as a non-pathogenic organism in humans and other animals. However, it can become pathogenic when the immune system is compromised due to bacterial, viral, fungal, or other parasitic infections, as well as systemic conditions, leading to symptomatic blastocystosis. Methods. Fecal samples were collected from patients at the University Hospital of Campania “Luigi Vanvitelli” and Cotugno Hospital in Naples. Among these samples, those that tested positive for *Blastocystis* spp. and were associated with other microbial infections were further analyzed. Bacterial co-infections were identified using immunochromatographic tests (ICTs) and matrix-assisted laser desorption/ionization time-of-flight mass spectrometry (MALDI-TOF MS). Viral infections were detected using chemiluminescent immunoassay (CLIA), while fungal infections were diagnosed through microscopic examination and molecular biology techniques. Additionally, co-infections with other parasites were identified through microscopic analysis after Ridley’s concentration and Giemsa staining (O&P). Results. Out of the 2050 stool samples collected, 121 were positive for *Blastocystis* spp., of which 75 were associated with other infections. We identified the vacuolar form in patients co-infected with bacteria (*n* = 22), viruses (*n* = 30), fungi (*n* = 3), and other parasites (*n* = 20). Conclusions. Our findings indicated a higher incidence of the vacuolar form of *Blastocystis* spp. in symptomatic and immunocompromised patients, suggesting that a weakened immune system may increase the risk of contracting Blastocystis and other microbial infections.

## 1. Introduction

Protozoan infections present various clinical symptoms, including fever, acute watery diarrhea, nausea, abdominal pain, vomiting, and dehydration. The main risk factors for their global prevalence include uncontrolled population growth, low levels of education and sanitation, climate change, and poor nutrition. *Blastocystis* spp. are common protozoans of the large intestine, causing blastocystosis, and their prevalence ranges from 7 to 20% in developed countries and 40–60% in developing countries [1,2]. Despite their origin dating back to the early part of the 20th century, their potential pathogenicity is still controversial: *Blastocystis* spp. are usually localized as a commensal in the intestine, where they feed on bacteria and food residues necessary for growth [3]; however, some strains are characterized by certain pathogenicity causing clinical signs and symptoms like abdominal pain, bloating, flatulence, traveler’s diarrhea, rectal bleeding, and irritable bowel syndrome, with extraintestinal cutaneous manifestations [4,5]. According to the World Health Organization (WHO), *Blastocystis* spp. are classified as neglected pathogens responsible for one of the twenty-one neglected tropical diseases (NTDs) [6,7]. The taxonomic classification of this protozoan is uncertain, but recent studies using sequencing of multiple conserved genes suggest that *Blastocystis* spp. belongs to the *Stramenopiles* group of protists.

Currently, 22 subtypes (STs) have been identified based on nucleotide variations in the rRNA gene [8]. Among these, *Blastocystis hominis* is the species found in human intestines. The predominant subtypes affecting humans include ST1, ST12 [9,10], and ST14, the latter described in 2020 by Khaled et al. Subtypes ST1, ST2, ST3, and ST4 are common in Europe. Notably, ST1, ST2, and ST3 are equally prevalent among healthy and sick individuals, whereas ST4 is epidemiologically linked to acute diarrhea and chronic conditions, such as irritable bowel syndrome, associated with enhanced biomarkers of gut inflammation.

The heterogeneity of *Blastocystis* spp. accounts for their variable virulence, influenced by factors such as protease secretion, damage to cellular barriers due to actin filament rearrangement, and the production of inflammatory cytokines by colon cells. The infectious forms of *Blastocystis* spp., likely the cyst and vacuolar forms, are presumed to be transmitted via the fecal–oral route, although they have not yet been definitively identified. The host ingests the cyst through water or food contaminated with fecal material. Once inside the large intestine, the cyst develops, progressing through various stages before being expelled into the environment. In the environment, the cyst can survive in water for up to one month at 25 °C and up to two months at 4 °C [3,11]. According to a study by F. Beghini et al., *Blastocystis* spp. are common components of the human gut microbiome [12]. However, they are also found in patients with dysbiosis, which is associated with conditions such as colorectal cancer and Crohn’s disease. Additionally, several studies have indicated that *Blastocystis* spp. are present in immunocompromised individuals, with a prevalence of 30 to 38% in developed countries. This prevalence suggests that they may be an important risk factor, particularly in cases involving viral co-infections, especially among patients infected with human immunodeficiency virus (HIV) [13,14,15].

*Blastocystis* spp. have also been identified alongside bacterial infections in the gastrointestinal tract. In patients experiencing diarrhea caused by *Clostridium difficile*, as noted by L. Deng et al. [16], the ST7 subtype of Blastocystis was predominant. Similarly, in an Italian study conducted in northeastern Italy [17], a large number of positive cases for the cagA factor of *Helicobacter pylori* were observed, highlighting the importance of screening for parasitic infections in such patients.

Further studies have confirmed the co-infection of *Blastocystis* spp. with other pathogens such as *Cryptosporidium* spp., *Toxocara* spp. [18], *Giardia* spp. [19], and *Entamoeba* spp. The presence of these other pathogens in the gastrointestinal tract can promote blastocystosis, as the commensal organisms can proliferate and reach higher concentrations in the intestine [20]. As demonstrated by Pielok et al. [18], subjects infected with oocysts of *Cryptosporidium* spp. [18] who were also co-infected with *Toxocara* spp. showed a significant presence of blastocystosis, which was further corroborated by cases of giardiasis described by Sánchez et al. Infections caused by bacteria, viruses, and parasites can increase the prevalence of blastocystosis and its most virulent subtype, leading to alterations in gut microbiota. In this context, Blastocystis can act as both a pathogen and a virulent agent. In this work, we analyzed the prevalence of blastocystosis in two hospital settings in southern Italy. Additionally, we reported cases of co-infection with various pathogens, including bacteria, viruses, fungi, and parasites.

## 2. Materials and Methods

### 2.1. Sample Collection

A total of 2050 stool samples were collected from the Complex Operative Unit (U.O.C) of Virology and Microbiology of two hospitals in Naples, including the University of Campania “L. Vanvitelli” and Cotugno Hospital. Sampling and identification were conducted between January 2022 and January 2024, following the recommended national and international guidelines [21,22]. Three samples from three different and non-consecutive days were collected from each patient with suspected bacterial, viral, fungal, or parasitic gastroenteritis.

### 2.2. Direct Microscopic Fresh Examination of Fecal Samples

A few grams of feces was diluted in 1–2 mL NaCl 0.9%; a drop of the diluted sample was placed on a glass slide and microscopic observation was immediately performed to search for the presence of parasites by observing motile structures (cilia or flagella) and the movements of certain parasites (amoebae or larvae). Another direct examination included preparation with Dobell’s solution to stain glycogen vacuoles and nuclei of cysts present.

### 2.3. Microscopic Examination After Ridley’s Concentration Method

Samples analyzed by Ridley’s method were fixed and concentrated with a Para-Pak® CON-Trate® System stool concentration kit (Meridian Bioscience, Cincinnati, United States). Four distinct phases were visible after the concentration of samples: a layer of ethyl acetate or solvent, a layer of feces debris, a slightly colored aqueous layer (formalin), and a sediment layer. This last phase was collected, and the sample was observed by microscopy.

### 2.4. Detection of Bacterial Co-Infections

#### 2.4.1. Clostridium Difficile Detection by GDH Immuno-Chromatographic Test: Tox A and Tox B

The stool samples were screened for antigens and toxins (Tox A and Tox B), using the Immunocard *Clostridium difficile* GDH (Meridian Bioscience, Cincinnati, OH, USA) according to the manufacturer’s instructions [22]. Briefly, the diluent sample and enzyme conjugate were added together with liquid stool. The specimen was mixed and then transferred to the card. After incubation for 15 min at room temperature, the substrate was added, and results were read in 10 min.

#### 2.4.2. *Helicobacter Pylori* Antigen Detection by Immuno-Chromatographic Test

A *Helicobacter pylori* Antigen Rapid Test Cassette (Alltest, Hangzhou, China) was used. Labeled antibodies attached to a nitrocellulose membrane were able to recognize and bind to the antigens present in the fecal sample, resulting in the formation of a colored band.

#### 2.4.3. *Salmonella spp. Detection*

One gram of fecal sample was inoculated in Selenite Broth Base broth (Biolife Italiana, Monza, Italy) and incubated at 35 ± 2 °C for 18–24 h. Subsequently, the turbidity of the broth was assessed, and 1 μL was plated on Hektoen Enteric Agar (H.E.A.; Becton Dickinson, Franklin Lakes, NJ, USA) and Salmonella and Shigella Agar (SS; Becton Dickinson). The plate was incubated at 35 ± 2 °C for 18–24 h, and mass spectrometry using matrix-assisted laser desorption/ionization (MALDI-TOF, Bruker, Biellerica, MA, USA) was executed (KIT Salmonella typhi H Micro 5 × 10 mL; LTA, Bussero, Milano, Italy).

### 2.5. Detection of Viral Co-Infections

The viral co-infections were detected by the LIAISON XL (Diasorin S.P.A., Saluggia, Italy). Specific viruses were investigated, including hepatitis viruses A (HAV) and B (HBV); adenovirus (AdV); SARS-CoV-2; cytomegalovirus (CMV); herpes simplex virus type 1 (HSV-1) and type 2 (HSV-2); Epstein–Barr virus (EBV); rubella virus (RuV); influenza viruses A and B; human immunodeficiency virus (HIV).

### 2.6. Fungal Co-Infections by DNA Identification

Fungal co-infection was assessed based on direct microscopic examination, which revealed the characteristic mycetic pseudohyphae of *Candida* spp., and by detecting *Pneumocystis jirovecii* DNA using a PCR assay (ELITE INGENIUS®-ELITechGroup Molecular Diagnostics, Torino, Italy) from a specific bronchoalveolar lavage sample, integrating extraction, amplification, and results interpretation.

### 2.7. Parasitic Co-Infections by Immuno-Chromatographic Tests and Molecular Confirmation

*Chilomastix mesnili*, *Dientamoeba fragilis*, *Endolimax nana*, *Entamoeba coli*, *Entamoeba histolytica*/*dispar*, *Enterocytozoon bieneusi*, *Giardia duodenalis*, and *Schistosoma mansoni* infections were detected by microscopy analysis as previously described. In addition, two kits were used for *Giardia* spp. identification: Cryptosporidium and Giardia Card Plus (Mascia Brunelli; Monza, Italy) and immunofluorescence assay (IFA) with Merifluor® Cryptosporidium/Giardia (Meridian Bioscience). Molecular analysis was also conducted by combining the PCR and microarray procedure with the Novadiag molecular biology system (Hologic, Marlborough, MA, USA).

### 2.8. DNA Extraction, PCR Amplification, and Microarray

The Novodiag molecular biology system (Hologic) was used for the detection of nucleic acids of the most common parasites, including *Ancylostoma duodenalis*, *Ascaris lumbricoides*, *Balantidium coli*, *Blastocystis* spp., *Diphyllobothrium latum*, *Clonorchis sinensis*, *Opisthrochis* spp., *Cryptosporidium* spp., *Cyclospora cayetanensis*, *Cystoisospora belli*, *Dientamoeba fragilis*, *Entamoeba histolytica*, *Enterobius vermicularis*, *Fasciola* spp., *Giardia intestinalis*, *Fasciolopsis buski*, *Hymenolepis nana*, *Schistosoma mansoni*, *Strongyloides stercoralsis*, *Taenia saginata*, *Taenia solium*, *Taenia asiatica*, and *Thricuris* spp. The procedure included the adsorption of a certain amount of feces via the eSwab; then, 1 mL of the fecal solution was inoculated into a tube containing agarose beads, and mechanical lysis of cells was carried out by the MagNALyser (Roche Diagnostics, Indianapolis, IN, USA). The cartridge containing specific PCR sequence primers was filled with 600 μL of the lysed cells, and PCR was performed according to manufacturer’s instructions (94 °C for 4 min; 95 °C for 15 s, 60 °C for 15 s, 72 °C for 30 s, repeated for 30 cycles; 72 °C for 5 min). Finally, the amplified sequences were paired with specific sequence microarrays for a qualitative result (absence/presence of parasitic DNA).

### 2.9. Statistical Analysis

The IBM SPSS software (version 22.0; IBM SPSS Inc., New York, NY, USA) was utilized for data analysis. The data were summarized and described using frequencies and proportions. For categorical variables, descriptive statistics were presented as counts and percentages. The chi-square test was used to compute *p*-values, with a significance threshold set at <0.05.

## 3. Results

### 3.1. Prevalence of Blastocystis spp. by Microscopic Detection

**Figure 1:**
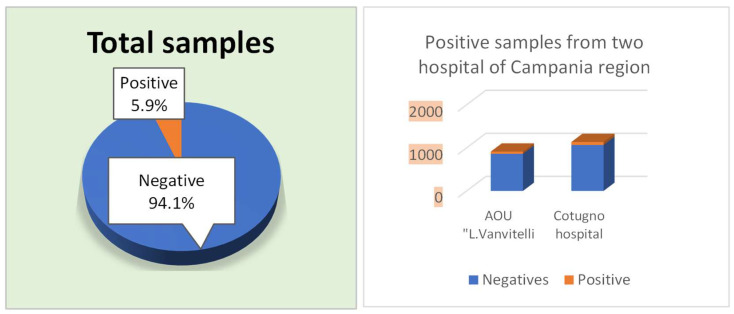
Total samples, Negative and Positive from AOU “L. Vanvitelli” and Cotugno Hospital. From 2050 samples, 5.9% positives were identified through fresh microscopic analysis after Ridley’s concentration and Giemsa staining (O&P).

During the period from January 2022 to January 2024, 2050 stool samples were collected in the two hospitals of the Naples area. In detail, 913 (44.5%) samples were isolated from the AOU of the University Hospital of Campania “Luigi Vanvitelli” while 1137 (55.5%) samples were collected from Cotugno Hospital (Figure 1). Among them, 121 (5.9%) were positive for *Blastocystis* spp.: 51 belonging to the AOU of the University Hospital of Campania “L. Vanvitelli” and 70 patients from Cotugno Hospital.

**Figure 2:**
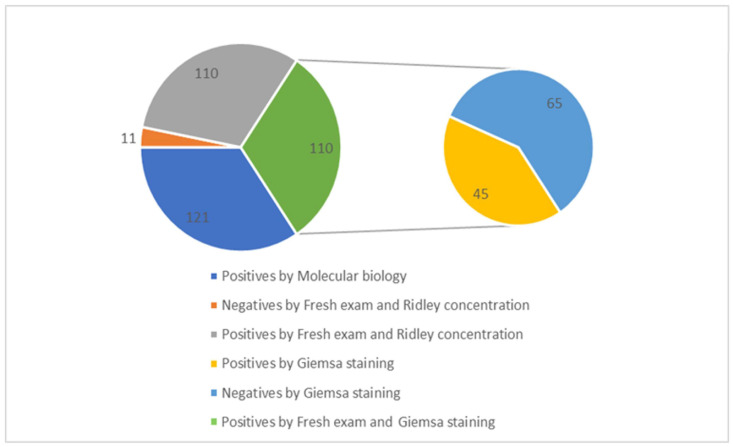
Correlation between total positive samples for *Blastocystis* spp. and Giemsa staining.

In contrast, 55.5% of the samples (*n* = 1137) came from Cotugno Hospital, which had 70 positive cases. Additionally, a further identification was performed to test the positive samples for *Blastocystis* spp. using Giemsa staining. A total of 121 samples were analyzed for *Blastocystis* spp. using molecular biology techniques, and it was confirmed that 45 of these samples were positive. Specifically, out of the 121 samples, 110 tested positive based on fresh examination and Ridley concentration methods. Among these, 45 samples were also positive when examined using Giemsa staining: 15 from the AOU of the University Hospital of Campania “Luigi Vanvitelli” and 30 from Cotugno Hospital. Additionally, 65 samples were found to be negative using the Giemsa staining method. This discrepancy is because Giemsa staining is less sensitive compared to microscopic observation following fresh examination, Ridley concentration, PCR, and microarray detection (Figure 2).

**Figure 3:**
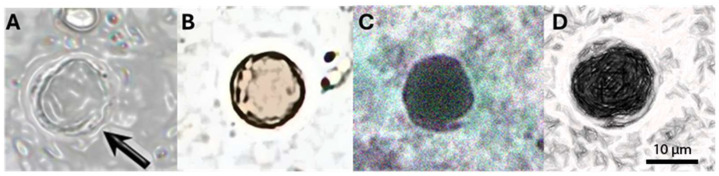
The most representative forms of *Blastocystis* spp. (**A**) The vacuolar form with a central vacuole and peripheric nuclei and cytoplasm; (**B**) The granular form with the presence of granulation; (**C**) The ameboid form with rgw presence of pseudopodia by the cytoplasm; (**D**) The cystic form with small vacuoles, granulation, and peripherical nuclei.

Among the samples that tested positive for *Blastocystis* spp., at least four major forms were identified through microscopic examination of fresh specimens. As shown in Figure 3, we observed the vacuolar form (Figure 3A), representing the most common form present in the culture and feces of the host. It was spherical and with a variable diameter (2–200 μm, average 4–15 μm). The vacuole occupied most of the cell, while the cytoplasm and nuclei were arranged in a ring at the periphery. As shown in Figure 3B, the granular form with a vacuolar shape is characterized by granulations in the cytoplasm; an amoeboid form (Figure 3C) was rarely observed, characterized by one or more extensions of the cytoplasm (pseudopodia). The cystic form (Figure 3D) is frequently found in the feces and is characterized by variable morphology (spherical to oval) and small size (2–5 μm). A multilayered wall surrounded the cyst and contained small vacuoles, glycogen granules, and one to four nuclei.

### 3.2. Detection of Co-Infecting Pathogens

**Figure 4:**
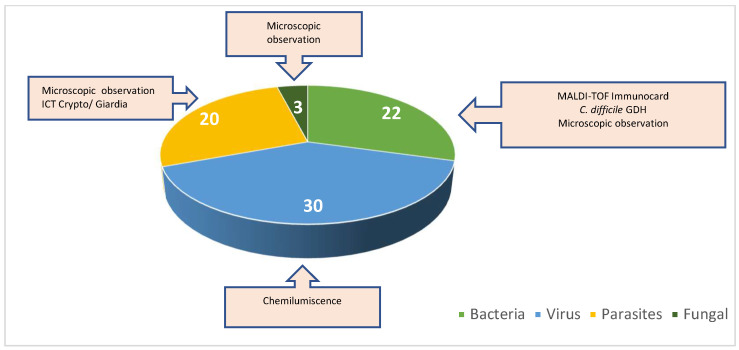
Percentage of co-infections with bacteria, viruses, parasites, and fungi.

In addition to detecting *Blastocystis* spp. in the analyzed samples, we also investigated the presence of co-infections among the positive cases. A total of 121 samples were examined for co-infections: 75 samples tested positive for co-infections, while 46 samples tested negative. Among the co-infected samples, 22 (29%) showed bacterial co-infections, 30 (40%) exhibited viral co-infections, 3 (4%) were characterized by fungal co-infections, and 20 (27%) had parasitic co-infections (Figure 4).

#### 3.2.1. Bacterial Co-Infection

**Figure 5:**
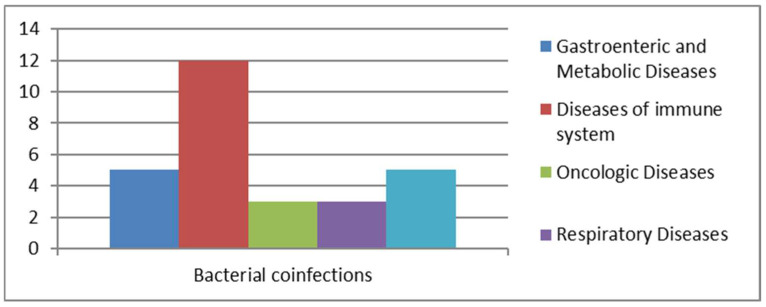
Bacterial co-infections.

We analyzed cases of *Blastocystis* spp. infections associated with bacterial co-infections (Figure 5).

Among the pathogens identified in samples with bacterial co-infections were both Gram-positive and Gram-negative bacteria capable of colonizing various regions of the human body, particularly the urinary and gastrointestinal tracts. Out of 22 positive cases, most showed co-infections with members of the Enterobacteriaceae family (*n* = 10) (Table 1). A smaller number of cases were caused by *Helicobacter pylori* (*n* = 3) and *Clostridium difficile*. Other pathogens were identified less frequently, including *Campylobacter* (*n* = 1), *Klebsiella pneumoniae* (*n* = 1), *Salmonella* spp. (*n* = 1), *Plesiomonas shigelloides* (*n* = 1), and *Treponema pallidum* (*n* = 1). The presence of pathogenic bacteria in the gastrointestinal tract, along with immunocompromisation of the immune system, favors the proliferation of commensal protozoa like *Blastocystis* spp. [18,20]. The total number of patients with bacterial co-infections was 22, which included 3 oncology patients, 3 patients with respiratory diseases, 5 transplant recipients, 5 individuals with gastrointestinal and metabolic issues, and 12 patients suffering from immune system disorders. Among these 12 patients, 2 also had metabolic diseases, while another 2 were oncology patients, and 2 were affected by bacterial pneumonia.

#### 3.2.2. Viral Co-Infections

**Figure 6:**
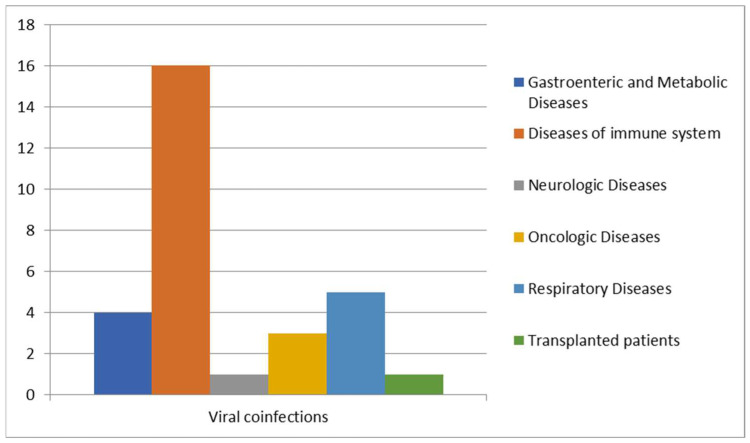
Correlation between viral infections and other diseases.

We further analyzed cases of *Blastocystis* spp. infection in patients with viral co-infections (Figure 6).

Viral co-infections were found in a higher number of the positive samples, with a prevalence of infections caused by members of the Herpesviridae family, retroviruses, and hepatitis viruses (Table 2).

A high prevalence of Blastocystis co-infections was observed in patients with problems of the immune system since people with HIV or with several other irreversible viral infections are the most susceptible [14,23]. Of the 30 patients positive for viral co-infections, like HAV, HBV, adenovirus, CMV, HSV-1 and HSV-2, RSV, EBV, influenzas A and B, Coxsackie virus, SARS-CoV-2, and HIV, 16 patients were affected by immune system problems [24], 5 patients by respiratory diseases, 4 patients by metabolic diseases, 3 patients were oncologic patients, 1 patient was affected by neurological disease, and 1 patient was transplanted.

#### 3.2.3. Fungal Co-Infections

**Figure 7:**
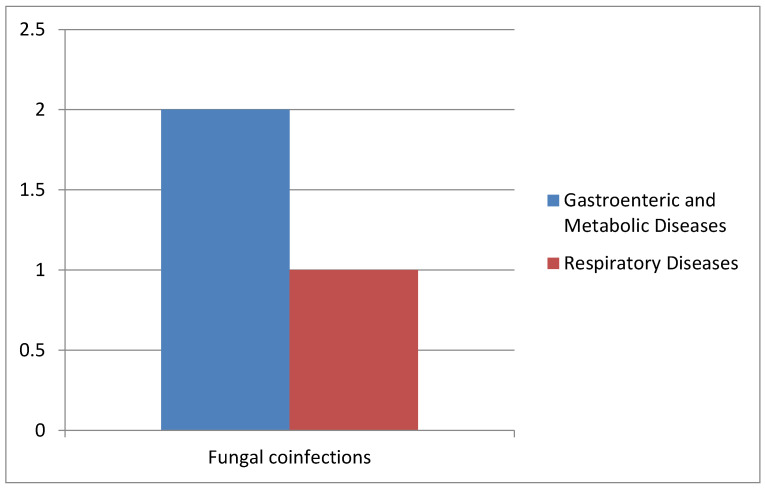
Correlation of *Blastocystis* and fungal co-infections with gastrointestinal and metabolic diseases and respiratory diseases.

In all collected samples, we also evaluated the presence of *Blastocystis* spp. and fungal infections (Figure 7).

In detail, two cases of co-infections with *Candida* spp. were observed in the AOU of the University Hospital of Campania “L. Vanvitelli”, and only one case affected by *Pneumocystis jirovecii* pneumonia was reported at Cotugno Hospital with the presence of *Pneumocystis* (Table 3).

#### 3.2.4. Parasitic Co-Infections

**Figure 8:**
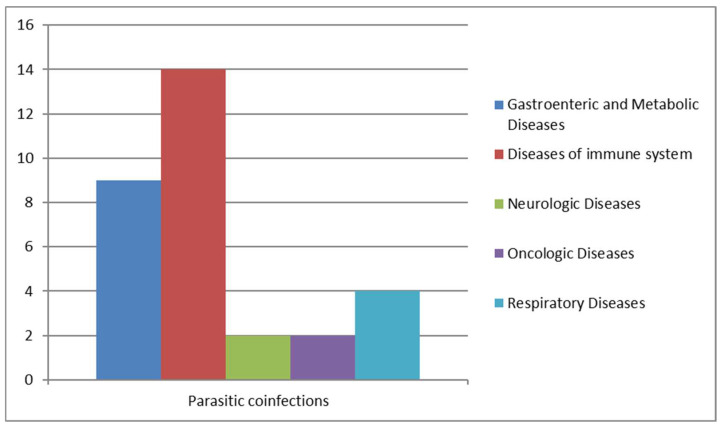
Parasitic co-infections correlated with other diseases.

We analyzed cases of Blastocystis infections associated with other parasitic infections: 14 patients with immune system problems, so the most susceptible and fragile to contract blastocystosis, 2 patients were also affected by metabolic diseases, 2 by respiratory diseases, 1 by neurologic diseases, and 1 was an oncologic patient (Figure 8).

As reported in Table 4, parasitic co-infection is detected in 30–69-year-old patients. An imposing incidence has been shown by parasitic co-infections: out of 121 positive samples, 20 were co-infected with parasites, like *Chilomastix mesnili*, *Dientamoeba fragilis*, *Entamoeba histolytica/dispar*, *Endolimax nana*, *Entamoeba coli*, *Enterocytozoon bieneusi*, *Giardia duodenalis*, and *Schistosoma mansoni*. Widely, we observed Entamoeba histolytica/dispar with the highest incidence, followed by *Giardia duodenalis* and *Schistosoma mansoni* (Table 4).

#### 3.2.5. Prevalence of *Blastocystis* spp.

We observed Blastocystis spp. and other microbial infections in 62 % of male and 38 % of female patients. Starting from 75 total positive patients with co-infections, four age groups were investigated (0–20; 21–40; 41–60; 61–80). The highest prevalence was observed at 21–40 years with 21% (*p*-value: 0804) and 41–60 years with 29% (*p*-value: 0048) (Table 5).

The incidence of *Blastocystis* spp. among various ethnic groups was also analyzed, with the highest values in the Italian population (88%). The most representative form was vacuolar in 88 samples (73%) and granular in 33 (27%). The presence of the first infectious form was significant in the patients with co-infections (Table 6). As reported in Table 6, *Blastocystis* spp. co-infections were more common in males (59%, *p*-value: 0.099) than females (41%, *p*-value: 0.548), with a high incidence (88%) in Italians, and the most frequent form was vacuolar (73%, *p*-value: <0.005).

#### 3.2.6. Molecular Analysis to Characterize *Blastocystis* spp.

To confirm the presence of positive cases of Blastocystis spp. and other parasites, molecular analysis was performed. A total of 121 patients (6%) tested positive using the Novodiag stool parasite system. Of these, 110 (91%) samples also tested positive upon retesting and after Ridley concentration, while 45 (37%) were confirmed positive by Giemsa staining (Table 7).

After comparison to standard reference methods, including direct microscopic examination of fresh samples, microscopic examination after Ridley concentration, and Giemsa (O&P) staining, the Novodiag test showed higher sensitivity and specificity, detecting even traces of parasitic DNA. The optimal approach would be a combination of both methods, with the Novodiag test used for initial screening of intestinal parasitic infections and the standard reference techniques used for confirming positive results.

By the comparison between standard retrieval techniques (O&P) for the search for parasites in feces and molecular diagnosis, it emerged that molecular biology and microarray are more sensitive (standard reference technique sensitivity: 87%/molecular analysis sensitivity: 100%). The specificities of the methods were the same (standard reference technique specificity: 100%, molecular analysis specificity: 100%). In fact, with 100% agreement, molecular biology confirms 121 positive cases of *Blastocystis* spp. versus 110 positive cases observed only by the microscopic exam (fresh examination and Ridley concentration) and only 45 positive results by a less specific method, Giemsa staining. A large number of positives by molecular biology are also associated with patients in therapy and with DNA in feces, detectable only by molecular analysis but not by direct search for vital microscopic forms.

## 4. Discussion

Our study investigated the prevalence of *Blastocystis* spp. infections and their correlation with other infectious diseases in two hospital settings in southern Italy: the University Hospital of Campania “Luigi Vanvitelli” and Cotugno Hospital. Sample collection took place from January 2022 to January 2024. We analyzed a total of 2050 samples using various methods by national and international guidelines. These methods included fresh examination of feces, Ridley’s concentration technique, Giemsa staining (O&P), and molecular biology approaches. Our findings highlighted the significance and prevalence of Blastocystis infections, occurring not only in endemic regions with poor sanitary conditions but also in industrialized countries. As demonstrated by Parija S.C. et al., the incidence of Blastocystis-related diarrhea has increased in recent decades, affecting both impoverished and Western globalized nations [25]. We identified a total of 121 positive samples for *Blastocystis* spp., with 22 co-infected with bacteria, 30 with viruses, 3 with fungi, and 20 with other parasites. Notably, a significant percentage of cases involved bacterial infections, particularly gastroenteric bacteria such as *Campylobacter*, *Clostridium difficile*, *Escherichia coli* (extended-spectrum β-lactamase), *Helicobacter pylori*, *Salmonella* spp., *Klebsiella pneumoniae*, *and Plesiomonas shigelloides*. Patients with these co-infections, as indicated by their medical history, exhibited a variety of symptoms related to intestinal diseases, including diarrhea (particularly traveler’s diarrhea), abdominal pain, gastrointestinal discomfort, bloating, flatulence, rectal bleeding, and irritable bowel syndrome.

Some patients exhibited extraintestinal skin manifestations. In contrast, individuals solely infected with *Blastocystis* spp. generally showed fewer symptoms. A retrospective data analysis was conducted to explore the correlation between *Blastocystis* spp. and other microorganisms. The analysis revealed a significant positive correlation between the presence of *Blastocystis* spp. and bacterial and viral infections (*p*-value: <0.05). This suggests that changes in the gastrointestinal microbiota or systemic viral infections are significantly associated with Blastocystis in the gut. Moreover, we observed that patients with co-infections more frequently exhibited vacuolar forms of *Blastocystis* spp., with cyst counts exceeding 20 per microscopic field. These patients displayed more pronounced clinical symptoms and had stool types classified as 6–7 on the Bristol stool scale. It is well established that co-infections typically lead to worse overall health outcomes, as they often pose greater treatment challenges. For example, patients who are co-infected with fungal pneumonia (*Pneumocystis jirovecii*), seasonal respiratory viruses, tuberculosis, and COVID-19 frequently experience intensified dysbiosis symptoms, such as diarrhea, due to compromised immune systems. However, our study did not find a significant correlation between *Blastocystis* spp. and fungal infections (*p*-value > 0.05). We recognize that this limitation may be due to the relatively small number of positive fungal samples. Interestingly, the granular form of Blastocystis was only observed in patients who were not co-infected with other pathogens. Several studies have suggested that *Blastocystis* spp. may have beneficial effects on the host by modulating the immune system. One potential mechanism is through the stimulation of mucus production via the cytokine IL-22, which may help alleviate colitis symptoms and improve gut health. Blastocystis plays a crucial role in modulating bacterial diversity and promoting the abundance of Clostridia species, which produce short-chain fatty acids, ultimately contributing positively to microbiota health [26,27]. Our research also confirmed that the vacuolar form is predominantly observed in the non-co-infected population, as 88 samples showing the vacuolar form were from this group, which appears to validate the proposed opportunistic pathogenic behavior of *Blastocystis* spp. This was previously emphasized in a study involving patients diagnosed with gastric and colorectal cancer where *Blastocystis* spp. was found in 11% of cancer-affected patients, who also exhibited diarrhea: the presence of blastocysts is a risk factor for the worsening of colorectal cancer by the alteration of the host immune response and by the increase in oxidative damage of the cells of the gastrointestinal tract; in this case, the subtype ST1 was the most prevalently detected in patients with colorectal cancer, with a significant risk of association (*p*-value: =0.004). In contrast, it was detected in only 2% of the control group [28] and also in another study involving HIV-positive patients that revealed that *Blastocystis hominis* infection decreased progressively with increasing CD4+ T-helper cells, while the risk of infection increased with higher HIV viral load. This confirms that an alteration of the immune system can cause a modification of the gastrointestinal tract and the microbiome; viral infections, like HIV, influence the production of cells of the immune response. In detail, T-helper cells regulate the immune response by producing and releasing cytokines that stimulate other cells to activate or suppress immune responses. HIV co-infection and the presence of Blastocystis not only aggravate the clinical signs of the commensal but also accelerate the progression of the acquired immunodeficiency syndrome [23,29,30]. In our study, we observed a correlation between the isolation of the vacuolar form of this parasite and the presence of bacterial, viral, and parasitic co-infections. The vacuolar form is most frequently found in patients with these co-infections. Anamnestic information shows that these patients are more likely to exhibit clinical signs of blastocystosis, such as diarrhea, flatulence, skin rashes, and irritable bowel syndrome. We believe that a diagnosis of *Blastocystis* spp. requires clinical attention and intervention, particularly in patients with a history of chronic or recurrent illness. The role of this parasite’s isolation is still unclear and warrants further investigation.

Additionally, our analysis of the prevalence of Blastocystis infection in two hospitals in the Campania region indicates that the symptomatology of Blastocystis infection cannot be fully explained by the presence of different subtypes or other microbial co-infections. This highlights the hypothesis that the host’s condition plays a crucial role in influencing the pathogenicity of *Blastocystis* spp. colonization. Considering the higher prevalence in younger patients, it would be worthwhile to evaluate the association between Blastocystis infection and intestinal microbiota or immunological conditions, as this could enhance our understanding of the parasite’s ability to penetrate the host.

## 5. Conclusions

Our study, conducted in two hospital settings in the Campania region, provides valuable insights into the incidence of infection and co-infection of *Blastocystis* spp. with other pathogens. We detected a total of 121 positive cases. A significant correlation was found between *Blastocystis* spp. and both bacterial and viral infections, suggesting that alterations in the gastrointestinal microbiota or systemic viral infections may be linked to the presence of this parasite. Patients with co-infections displayed more pronounced clinical symptoms, including diarrhea, abdominal pain, and gastrointestinal discomfort. They were also more likely to exhibit the vacuolar form of *Blastocystis* spp., which is frequently associated with pathogenicity. Our findings emphasize the importance of clinical attention when diagnosing *Blastocystis* spp., particularly in patients who experience chronic or recurrent gastrointestinal symptoms. Further investigation is necessary to enhance diagnostic and therapeutic strategies for managing co-infections and related health complications.

## Figures and Tables

**Table 1:** Bacterial co-infection in the University Hospital “Luigi Vanvitelli” and Cotugno Hospital.

AOU of University Hospital of Campania “Luigi Vanvitelli”		
Sex	Age	Species
Male	72	*Helicobacter pylori*
Male	14	*Clostridium difficile*
Male	9	*Enterobacteriaceae*
Female	64	*Helicobacter pylori*; *Clostridium difficile*
Female	4	*Salmonella* spp.
Female	25	*Escherichia coli Extended-Spectrum-β-Lactamase*
Female	65	*Enterobacteriaceae*
Female	46	*Enterobacteriaceae*
Female	4	*Enterobacteriaceae*; *Klebsiella pneumoniae*
Male	61	*Enterobacteriaceae*
Male	59	*Enterobacteriaceae*; *Clostridium difficile*
Female	76	*Clostridium difficile*
Male	14	*Enterobacteriaceae*; *Campylobacter*
Male	6	*Enterobacteriaceae*
Male	67	*Enterobacteriaceae*
Female	55	*Enterobacteriaceae*
**Cotugno Hospital**		
Female	41	*Helicobacter pylori*
Female	36	*Enterobacteriaceae*
Female	36	*Plesiomonas shigelloides*
Male	38	*Treponema pallidum*

**Table 2:** Viral co-infection in University Hospital “Luigi Vanvitelli” and Cotugno Hospital.

University Hospital of Campania “Luigi Vanvitelli”		
Sex	Age	Species
Male	72	HAV; HBV
Male	14	HAV
Male	59	HBV
Male	4	Adenovirus; CMV; HBV
Female	45	HSV-1; HSV-2; RuV
Female	4	EBV; influenzas A and B
Male	58	CMV
Male	64	HAV
Female	25	Coxsackie
Female	4	HSV-1; HSV-2; RuV
Male	30	HBV
Male	42	HBV
**Cotugno Hospital**		
Female	36	EBV/SARS-CoV-2
Male	53	HIV
Male	43	HBV
Male	60	HIV
Female	61	CMV; SARS-CoV-2
Female	59	CMV
Female	44	HBV
Female	18	EBV
Female	38	HIV
Female	39	HIV
Male	18	HBV; CMV
Male	18	HIV; CMV
Female	36	HBV
Male	37	HIV
Male	51	CMV; HAV; HBV
Male	14	HBC
Female	43	HAV; HIV

**Table 3:** Cases of fungal co-infection in the University Hospital “L. Vanvitelli” and Cotugno Hospital.

University Hospital of Campania “Luigi Vanvitelli”		
Sex	Age	Species
Male	74	*Candida* spp.
Female	10	*Candida* spp.
Cotugno Hospital		
Male	58	*Pnuemocystis jirovecii*

**Table 4:** Cases of parasitic co-infection in the University Hospital “L. Vanvitelli” and Cotugno Hospital.

University Hospital of Campania “Luigi Vanvitelli”		
Sex	Age	Species
Male	33	*Chilomastix mesnili*
Male	33	*Dientamoeba fragilis*
Female	39	*Giardia duodenalis*
Female	46	*Entamoeba histolytica/dispar*
Male	30	*Schistosoma mansoni*
**Cotugno Hospital**		
Female	57	*Entamoeba histolytica/dispar*, *Endolimax nana*, and *Entamoeba coli*
Female	44	*Entamoeba histolytica/dispar*
Female	46	*Entamoeba histolytica/dispar*
Male	39	*Giardia duodenalis*
Male	52	*Giardia duodenalis*
Male	38	*Entamoeba histolytica/dispar*
Male	38	*Giardia intestinalis*, *Chilomastix mesnili*, and *Dientamoeba fragilis*
Male	40	*Giardia duodenalis*
Male	50	*Dientamoeba fragilis*
Male	69	*Dientamoeba fragilis*
Male	61	*Enterocytozoon bieneusi* and *Giardia intestinalis*
Male	47	*Entamoeba histolytica/dispar*
Male	30	*Schistosoma mansoni*
Male	18	*Schistosoma mansoni*
Male	64	*Dientamoeba fragilis*

**Table 5:** Incidence of *Blastocystis* spp., analyzing specific age ranges of patients.

Age of Patients with Co-Infections				
Age groups	No. total of positive With co-infections	No. of positive With co-infections for age	Incidence (%)	*p* value
0–20	75	16	21	0.708
21–40	75	22	29	0.804
41–60	75	23	31	0.048
61–80	75	14	19	0.420

**Table 6:** Detection of the incidence of *Blastocystis* spp. co-infections in patients, specifying gender, nationality, and form. N/A: not available.

Characteristics	Total No. of Positives Examined for *Blastocystis* spp.	No. of Positives with Co-Infections	Incidence (%)	*p*-Value
Gender				
Male	78	44	59	0.099
Female	43	31	41	0.548
Ethnicity				
Ghana	5	5	4	N/A
India	6	4	5	N/A
Morocco	4	3	3	N/A
Italy	106	106	88	N/A
Form of *Blastocystis* (n =121)				
Granular	33	27	<0.005	
Vacuolar	88	73	<0.005	

**Table 7:** Concordance of epidemiological data.

	Positive Samples	Negative Samples	% Agreement
Fresh exam and exam after the Ridley concentration	110	11	91
Giemsa staining	45	76	37.2
Molecular analysis	121	0	100
	**Sensitivity**	**Specificity**	
Standard reference technique (O&P)	87%	100%	Standard reference technique (O&P)
Molecular analysis	100%	100%	Molecular analysis

## Data Availability

Further inquiries can be directed to the corresponding author.
